# Downregulation of zinc finger protein 71 in laryngeal squamous cell carcinoma tissues and its potential molecular mechanism and clinical significance: a study based on immunohistochemistry staining and data mining

**DOI:** 10.1186/s12957-022-02823-8

**Published:** 2022-11-11

**Authors:** Fang-Cheng Jiang, Jia-Yuan Luo, Yi-Wu Dang, Hui-Ping Lu, Dong-Ming Li, Zhi-Guang Huang, Yu-Lu Tang, Ye-Ying Fang, Yu-Xing Tang, Ya-Si Su, Wen-Bin Dai, Shang-Ling Pan, Zhen-Bo Feng, Gang Chen, Juan He

**Affiliations:** 1 Department of Pathology, The First Affiliated Hospital of Guangxi Medical University, 6 Shuangyong RD, Nanning, Guangxi Zhuang Autonomous Region 530021 People’s Republic of China; 2 Department of Radiology, The First Affiliated Hospital of Guangxi Medical University, 6 Shuangyong RD, Nanning, Guangxi Zhuang Autonomous Region 530021 People’s Republic of China; 3 Department of Pathology, Liuzhou People’s Hospital, 8 Wenchang RD, Liuzhou, Guangxi Zhuang Autonomous Region 545006 People’s Republic of China; 4 Department of Pathophysiology, School of Pre-clinical Medicine, Guangxi Medical University, 6 Shuangyong RD, Nanning, Guangxi Zhuang Autonomous Region 530021 People’s Republic of China

**Keywords:** Zinc finger protein 71 (ZNF71), Laryngeal squamous cell carcinoma (LSCC), Immunohistochemistry (IHC) staining, Single-cell RNA sequencing (scRNA-seq), Molecular mechanism, Immune infiltration

## Abstract

**Background:**

The molecular mechanism of laryngeal squamous cell carcinoma (LSCC) is not completely clear, which leads to poor prognosis and treatment difficulties for LSCC patients. To date, no study has reported the exact expression level of zinc finger protein 71 (ZNF71) and its molecular mechanism in LSCC.

**Methods:**

In-house immunohistochemistry (IHC) staining (33 LSCC samples and 29 non-LSCC samples) was utilized in analyzing the protein expression level of ZNF71 in LSCC. Gene chips and high-throughput sequencing data collected from multiple public resources (313 LSCC samples and 192 non-LSCC samples) were utilized in analyzing the exact mRNA expression level of ZNF71 in LSCC. Single-cell RNA sequencing (scRNA-seq) data was used to explore the expression status of ZNF71 in different LSCC subpopulations. Enrichment analysis of ZNF71, its positively and differentially co-expressed genes (PDCEGs), and its downstream target genes was employed to detect the potential molecular mechanism of ZNF71 in LSCC. Moreover, we conducted correlation analysis between ZNF71 expression and immune infiltration.

**Results:**

ZNF71 was downregulated at the protein level (area under the curve [AUC] = 0.93, *p* < 0.0001) and the mRNA level (AUC = 0.71, *p* = 0.023) in LSCC tissues. Patients with nodal metastasis had lower protein expression level of ZNF71 than patients without nodal metastasis (*p* < 0.05), and male LSCC patients had lower mRNA expression level of ZNF71 than female LSCC patients (*p* < 0.01). ZNF71 was absent in different LSCC subpopulations, including cancer cells, plasma cells, and tumor-infiltrated immune cells, based on scRNA-seq analysis. Enrichment analysis showed that ZNF71 and its PDCEGs may influence the progression of LSCC by regulating downstream target genes of ZNF71. These downstream target genes of ZNF71 were mainly enriched in tight junctions. Moreover, downregulation of ZNF71 may influence the development and even therapy of LSCC by reducing immune infiltration.

**Conclusion:**

Downregulation of ZNF71 may promote the progression of LSCC by reducing tight junctions and immune infiltration; this requires further study.

**Supplementary Information:**

The online version contains supplementary material available at 10.1186/s12957-022-02823-8.

## Introduction

Laryngeal squamous cell carcinoma (LSCC) is one of the most common malignant tumors in the head and neck, with a high metastatic rate and recurrence rate [1]. At present, treatment methods include surgery, radiotherapy, and chemotherapy [2, 3]. Despite therapeutic progress, overall mortality remains approximately 50%, and the quality of life of patients is poor, especially for advanced patients [1, 4]. To date, there is no tool to predict the evolution of this type of cancer [5]. The molecular mechanism of LSCC also remains unclear. Therefore, many studies have been conducted to find biological indicators related to the invasion and metastasis of LSCC in recent years to provide a new direction for clinical treatment by elucidating the molecular mechanism.

Zinc finger protein (ZNF), the largest transcription factor (TF) family in the human genome, has an extraordinarily diverse set of functions, including DNA recognition, transcriptional activation, RNA packaging, apoptosis regulation, protein folding and assembly, and lipid binding [6]. In a recent study by Jen and Wang shows, ZNF influences cancer progression by abnormally expressing proteins [6]. As a member of the family, ZNF71 expression is associated with chemical sensitivity, and protein expression correlates with the prognosis of non-small cell lung cancer (NSCLC) [7]. Increased ZNF71 protein expression is positively related to a favorable prognosis in NSCLC [8]. Moreover, the expression of ZNF71 was lower in the high-risk group than in the low-risk group of osteosarcoma patients with poor prognoses [9]. However, to the authors’ knowledge, no study has proven the specific expression status and potential molecular mechanism of ZNF71 in LSCC.

Therefore, the present study aims to determine the specific expression level of ZNF71 in LSCC by analyzing a large of samples from multi-centers and in-house immunohistochemistry (IHC) staining results. In this study, single-cell RNA sequencing (scRNA-seq) results revealed the expression status in different subpopulations of patients with LSCC. Then, the potential molecular mechanism of ZNF71 in LSCC was explored by conducting enrichment analysis and predicting the immune-infiltration landscape.

## Materials and methods

### Evaluation of ZNF71 protein expression in LSCC tissues based on in-house immunohistochemistry staining

We purchased 33 LSCC tissue microarray samples (number HNT1021) and 17 normal laryngeal tissue microarray samples (number HNT1021) from Guilin Fanpu Biotech (Guangxi, China), and these samples were collected from the north of Guangxi. We also collected 12 non-cancerous squamous epitheliums of laryngeal tissues from the First Affiliated Hospital of Guangxi Medical University, China. These samples were collected from the south of Guangxi which includes 6 benign papilloma of laryngeal tissue samples and 6 normal laryngeal tissue samples. Our work was approved by the Ethics Committee of the First Affiliated Hospital of Guangxi Medical University (number 2021-KY-E-117). All study participants agreed to the study, and we received written informed consent from study participants. In total, we collected 33 LSCC samples and 29 non-LSCC tissue samples. Furthermore, we downloaded subcellular location images from the Human Protein Atlas (HPA) website (https://www.proteinatlas.org/ENSG00000197951-ZNF71/subcellular) to identify the subcellular location of the ZNF71 protein.

ZNF71 polyclonal antibody was purchased from biorbyt, and it is a rabbit-anti-human antibody (No. orb335284). We used the antibody for IHC staining (dilution 1:100) and operated strictly under the manufacturer’s instructions. The tissue slides had been formalin-fixed and paraffin-embedded, so we deparaffinized and rehydrated them. Then, we incubated them with 3% H_2_O_2_ for 5–10 min, rinsed them with distilled water, and soaked them in phosphate-buffered saline (PBS). We blocked the slides with the purchased ZNF71 antibody and washed them with PBS. Then, the tissue slides were re-stained, dehydrated, made transparency, and sealed. We performed these procedures at room temperature. Finally, two pathologists scored the final IHC tissue slides according to the staining intensity and quantity of positive cells (proportion of stained cells in tumor cells of the whole visual field). The scores of staining intensity followed the criteria: no staining/blue (point 0), weak staining/light yellow (point 1), moderate staining/tan (point 2), and strong staining/sepia (point 3). The scoring criteria was as follows: 0–5% (point 0), 6–25% (point 1), 25–50% (point 2), 50–75% (point 3), and 75–100% (point 4). The total IHC score was the product of the two scores; the final score for each slide was the average of the total IHC scores determined by each of the two pathologists. More specific information can be referenced in previous work by our team [10–12]. And the raw data for IHC staining is presented in Table S1.

We statistically analyzed and visualized the expression differences of ZNF71 in LSCC samples and non-LSCC samples through GraphPad Prism v8.2.1 software. *T* test was used here, and *p* value < 0.05 was considered that the results have statistical significance. And we plotted the receiver operating characteristic (ROC) curve and calculated the area under the curve (AUC) value through GraphPad Prism v8.2.1 software.

### Identification of ZNF71 mRNA expression in LSCC tissues based on data collected from multi-centers

We collected extensive LSCC and non-cancerous laryngeal tissue samples from several public databases, such as Gene Expression Omnibus (GEO), The Cancer Genome Atlas (TCGA), Sequence Read Archive (SRA), ArrayExpress, and Oncomine. The search terms, inclusion criteria, and exclusion criteria can be referenced in previous work by our team [11]. Finally, 12 datasets across 10 platforms were obtained. The specific information is presented in Table S2. We then removed the batch effect of datasets from the same platform and combined them via the sva package (https://bioconductor.org/packages/sva/) in RStudio v3.6.1. We logarithmically processed the expression values of ZNF71 which were not normalized via RStudio v3.6.1. We extracted the expression values of ZNF71 gene from these datasets for next calculation, and the data is presented in Table S3. We then calculated the standardized mean difference (SMD) value in the Stata v12.0 software using the expression values of ZNF71 gene. The fixed-effect model was used because *I*^2^ < 50%. ∣SMD∣ > 0 and *p* value < 0.05 were considered that the results have statistical significance. Through the Stata v12.0 software, we conducted sensitivity analysis to assess the stability of the results. Then, we used Begg’s and Egger’s test to test the publication bias of included cohorts. Here, *p* value > 0.05 was considered the included datasets have no publication bias. We also plotted the ROC curve and calculated the AUC value of the ROC curve via GraphPad Prism v8.2.1 software. We next plotted the summary ROC (sROC) curve using the expression values of ZNF71 and calculated the AUC of the sROC curve with Stata v12.0 software. Deek’s test was used to test the publication bias, and *p* value > 0.05 indicated no publication bias. We created a forest plot to describe the variance of sensitivity and specificity using the Stata v12.0 software. We also performed correlation analysis between ZNF71 expression and clinic-pathological parameters using TCGA_LSCC clinical data through GraphPad Prism v8.2.1 software.

### Detection of ZNF71 expression landscape based on single-cell RNA sequencing analysis

To explore the expression status in LSCC across different subpopulations, we collected a scRNA-seq dataset (GSE150321) and selected one untreated LSCC sample from the dataset named GSM4546858 for analysis [13]. We used a seurat process for scRNA-seq dataset analysis via the Seurat (https://cloud.r-project.org/package=Seurat) package in RStudio v3.6.1 [14]. We set filtering conditions for quality control, and after screening, all cells we remained these cells with numbers of genes exceeding 50 and numbers of mitochondrial genes less than 30%. We then standardized the total gene expression value of the remaining cells via the Seurat package in RStudio v3.6.1. And we scaled the data and analyzed the principal components. After dimensionality reduction using the Seurat package, we selected the first 20 principal components for subsequent analysis. Here, we used the *t*-distributed stochastic neighbor embedding (tSNE) method for dimension reduction and clustering. We used “FindClusters” function to divide LSCC cells with resolution = 0.5. Finally, we identified 15 clusters via the Seurat package in RStudio v3.6.1. We showed the logarithmically processed expression value of ZNF71 in each cell subpopulations and visualized it via the Seurat package in RStudio v3.6.1. We then annotated the cell type of each cluster according to the method provided by dataset contributors [13]. And we visualized it in RStudio v3.6.1.

### Enrichment analysis for PDCEGs and downstream target genes of ZNF71

Pearson correlation analysis was used to calculate the positive co-expressed genes of ZNF71 from 10 cohorts via the Pearson function in RStudio v3.6.1. The screening conditions were as follows: (a) *p* value < 0.01, (b) *r* (Pearson correlation coefficient) ≥ 0.4, and (c) repetition ≥ 2. After intersecting with the downregulated gene set, a total of 106 positively and differentially co-expressed genes (PDCEGs) was obtained and these PDCEGs are presented in Table S4. We calculated the SMD values for each gene to identify differentially expressed genes (DEGs) from 10 cohorts via the meta package (http://cran.r-project.org/web/packages/meta/index.html) in RStudio v3.6.1. We considered DEGs with criteria value of ∣SMD∣ > 0 and of *p* value < 0.05. Results are expected to be more reliable due to the multiple datasets included. Then, we constructed a protein–protein interaction network (PPI) to explore the relationships between those PDCEGs and annotated their biological function and signaling pathways through the STRING website (https://string-db.org/) [15]. We used the Cistrome Data Browser (DB) (http://cistrome.org/db), an online tool derived from chromatin immunoprecipitation and DNA sequencing (ChIP-seq) chromatin profiling assays, assay for transposase-accessible chromatin with high throughput sequencing (ATAC-seq), and DNA affinity purification sequencing (DNase-seq), to predict the downstream target genes of ZNF71 [16]. We set filtering conditions, namely a score greater than 0.1 and positive strand. After intersecting with the downregulated genes in LSCC, a total of 889 target genes was obtained. Then, we used Gene Ontology (GO) and Kyoto Encyclopedia of Genes and Genomes (KEGG) analysis to annotate the function and signaling pathway of these target genes via the ClusterProfiler package (http://bioconductor.org/packages/clusterProfiler/) in RStudio v3.6.1.

### Correlation analysis between ZNF71 expression and immune infiltration in LSCC

The ESTIMATE algorithm is a method for inferring the fraction of stromal and immune cells in tumor samples using gene expression signatures [17]. We calculated the ESTIMATE score of each TCGA_LSCC sample via the ESTIMATE package (https://r-forge.r-project.org/projects/estimate/) in RStudio v3.6.1. Then, we performed correlation analysis between the ESTIMATE score and ZNF71 expression in GraphPad Prism v8.2.1 software. To further explore the relationship between ZNF71 expression and tumor-infiltration lymphocytes (TILs), we used TISIDB to analyze it. TISIDB (http://cis.hku.hk/TISIDB) is a user-friendly web portal that pre-calculates the associations between any gene and immune features [18]. Then we calculated the association between ZNF71 expression and immune infiltration for the TCGA_LSCC samples.

### Statistical analysis

In this study, the software GraphPad Prism v8.2.1, Stata v.12.0, RStudio v3.6.1 were used for statistical analysis and results visualization. In GraphPad Prism v8.2.1 software, the *t* test was used for data that met both normal distribution and variance homogeneity parameters; the Wilcoxon test was used for other data. We used the interval estimation method to calculate the 95% confidence interval (95% CI). And *p* value < 0.05 was considered the results have statistical significance.

## Results

### ZNF71 was downregulated at the protein level in LSCC tissues

**Fig. 1:**
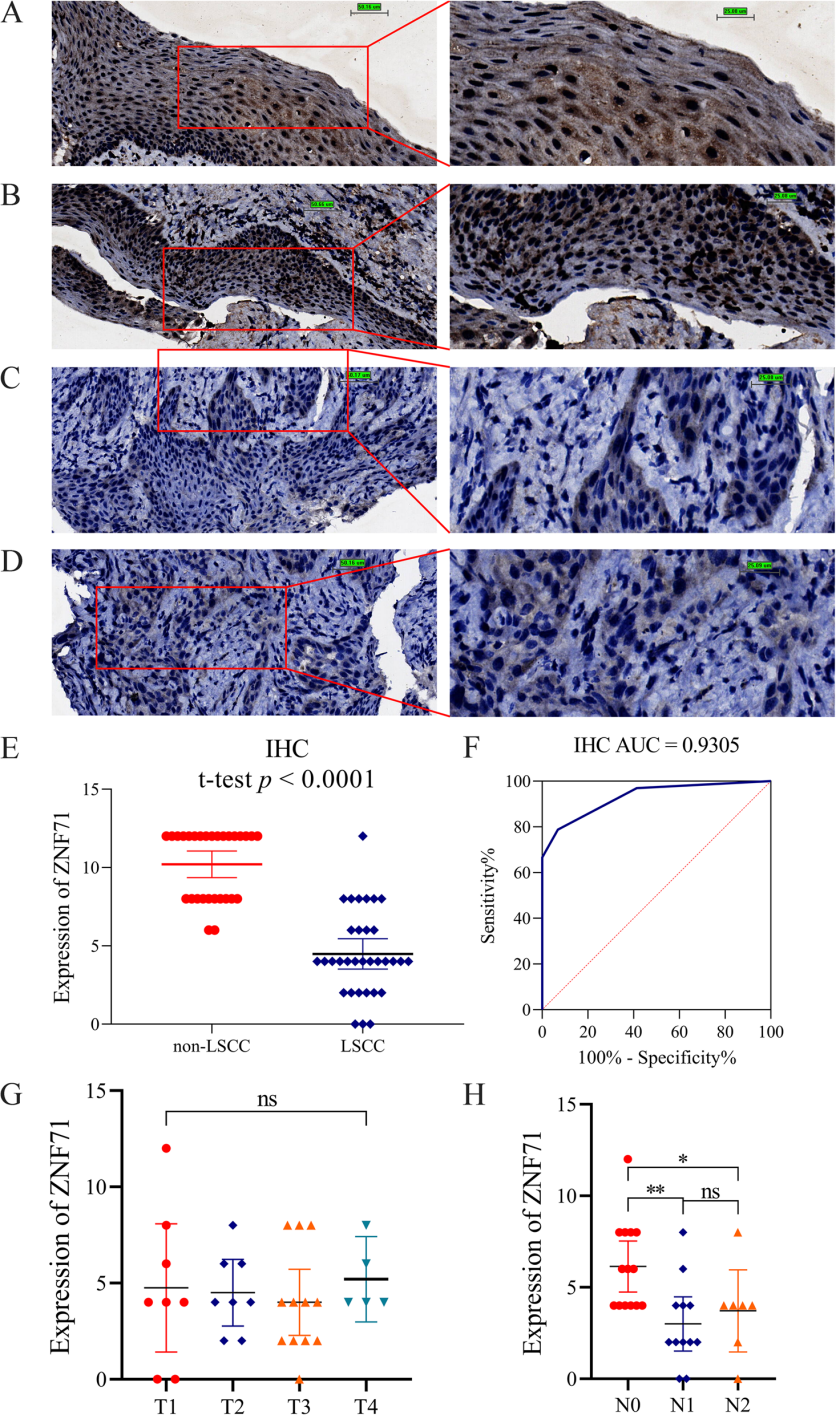
IHC staining of ZNF71 protein in laryngeal squamous cell carcinoma (LSCC) and non-LSCC tissues. **A**, **B** Expression level of ZNF71 protein in normal laryngeal tissues (left: ×200; right: ×400). **C**, **D** Expression level of ZNF71 protein in LSCC tissues (left: ×200; right: ×400). **E** Scatter plot of IHC score. **F** The ROC curve of ZNF71 expression. **G**, **H** Differentially expression analysis of ZNF71 protein between different T stage (**G**) and N stage (**H**). Note: ns: non-significant; *p < 0.05; **p < 0.01

According to the results from the HPA website, the ZNF71 protein is mainly localized to the nucleoli (Figure S1). In the IHC staining of non-cancerous laryngeal tissues, we observed that the staining of the nucleus was positive (Fig. 1A, B), while in the IHC staining of LSCC tissues, the staining of whole tissues was negative (Fig. 1C, D). To further quantify the results, we calculated the statistical difference between the IHC scores of the LSCC and non-LSCC tissues, and the results showed that the expression of ZNF71 was downregulated in LSCC tissues (*p* < 0.0001, Fig. 1E). And the ROC curve indicated that the ZNF71 protein has great discriminatory ability between LSCC and non-LSCC tissues (*p* < 0.0001, Fig. 1F). Moreover, the correlation analysis between the protein expression of ZNF71 and clinicopathologic parameters were conducted. The results showed us that patients with different T stage had no difference of ZNF71 expression (*p* > 0.05, Fig. 1G). However, patients with nodal metastasis had lower protein expression level of ZNF71 than patients without nodal metastasis (*p* < 0.05, Fig. 1H).

### ZNF71 was downregulated at the mRNA level in LSCC based on comprehensive analysis

**Fig. 2:**
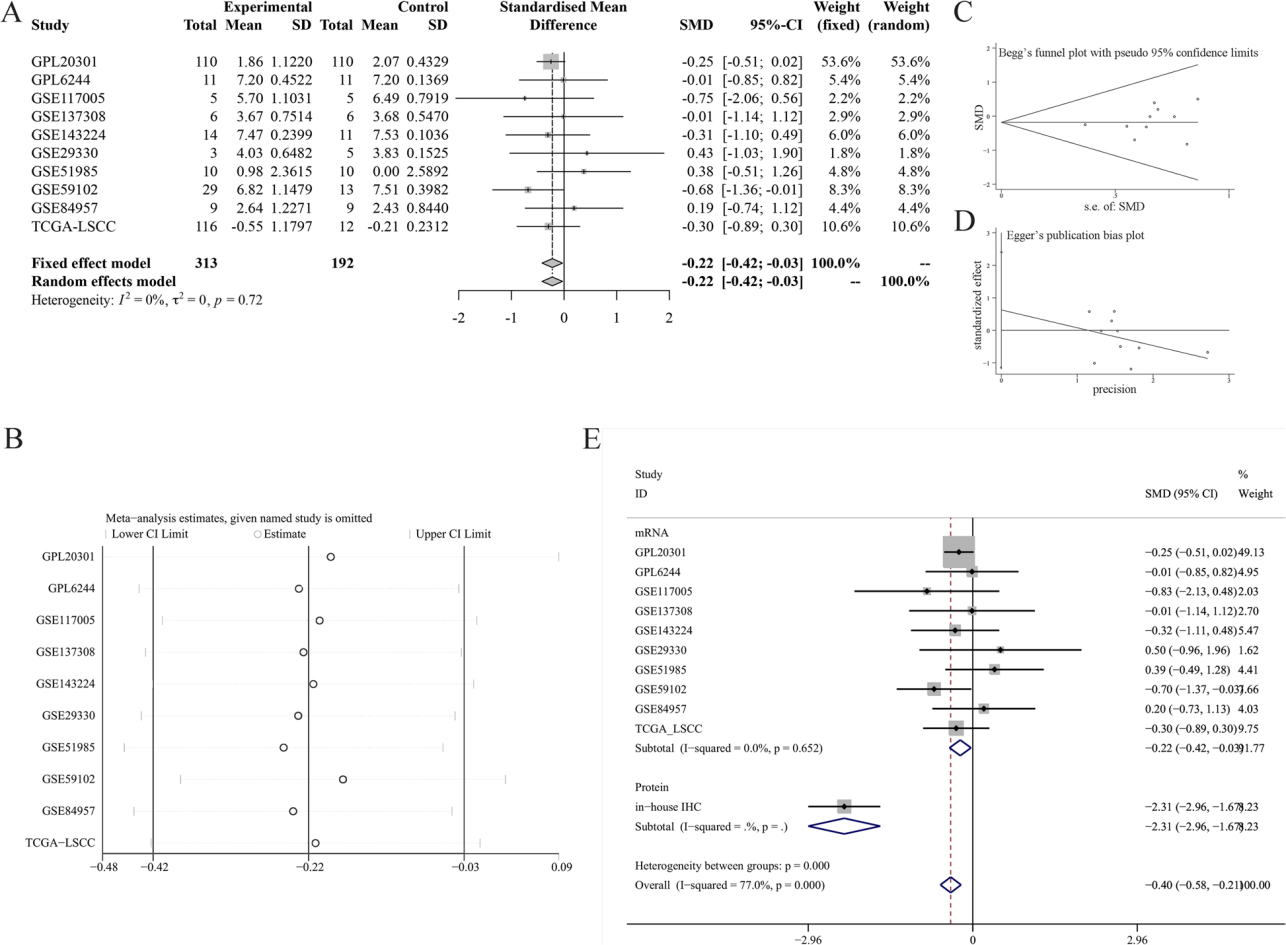
Identification of ZNF71 mRNA expression between LSCC and non-cancerous laryngeal tissues based on comprehensive analysis. **A** Forest plot of ZNF71 expression of included datasets. **B** Sensitivity analysis of included analysis; Begg’s test (**C**) and Egger’s test (**D**) for publication bias test of included datasets. **E** Forest plot of ZNF71 expression integrating the mRNA data and protein data of in-house IHC

We comprehensively analyzed the multiple datasets to clarify the exact expression status of ZNF71 in LSCC. The forest plot showed that ZNF71 was downregulated at mRNA level in LSCC (total SMD: −0.22, 95% CI, −0.42–−0.03, Fig. 2A). Sensitivity analysis (Fig. 2B) showed that the results from the included datasets were robust. We tested the publication bias, and the Begg’s test (*p* = 0.283, Fig. 2C) and Egger’s test (*p* = 0.438, Fig. 2D) showed that there was no publication bias. After comprehensive analysis, it was confirmed that the expression of ZNF71 was downregulated in LSCC tissues. Furthermore, we integrated the mRNA data and the protein data of in-house IHC, and we found the downregulation status of ZNF71 in LSCC tissues (total SMD: −0.40, 95% CI, −0.58–−0.21, Fig. 2E).

**Fig. 3:**
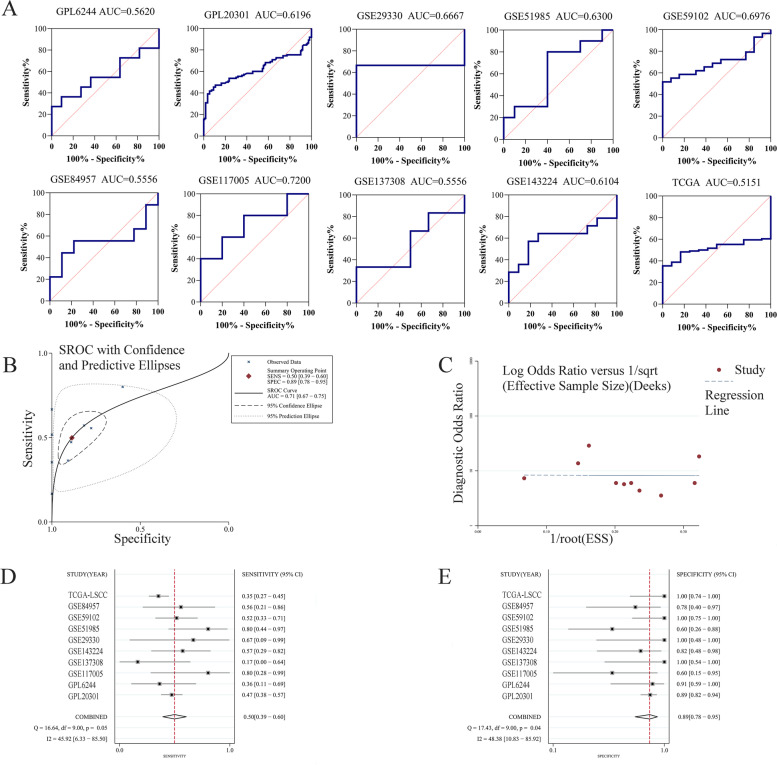
Discriminatory ability analysis of ZNF71 between LSCC and non-cancerous laryngeal tissues. **A** The ROC curve of ZNF71 in each included dataset. **B** Summary ROC curve of ZNF71. **C** Deek’s test for publication bias test; sensitivity (**D**) and specificity (**E**) analysis of ZNF71 expression in LSCC and non-LSCC samples based on included datasets

To explore the discriminatory ability of ZNF71 expression between LSCC and non-cancerous laryngeal tissues, we analyzed the ROC curves of all included datasets and calculated the AUC values (Fig. 3A). We further analyzed the sROC curve to describe this discriminatory ability more intuitively. The results of the sROC showed that ZNF71 has moderate discriminatory ability in LSCC (AUC = 0.71, 95% CI, 0.67–0.75, Fig. 3B). Deek’s test showed that the included datasets had no publication bias (*p* = 0.969, Fig. 3C). The total sensitivity of the included datasets was 0.50 (95% CI, 0.39–0.60, Fig. 3D), and the total specificity was 0.89 (95% CI, 0.78–0.95, Fig. 3E).

**Fig. 4:**
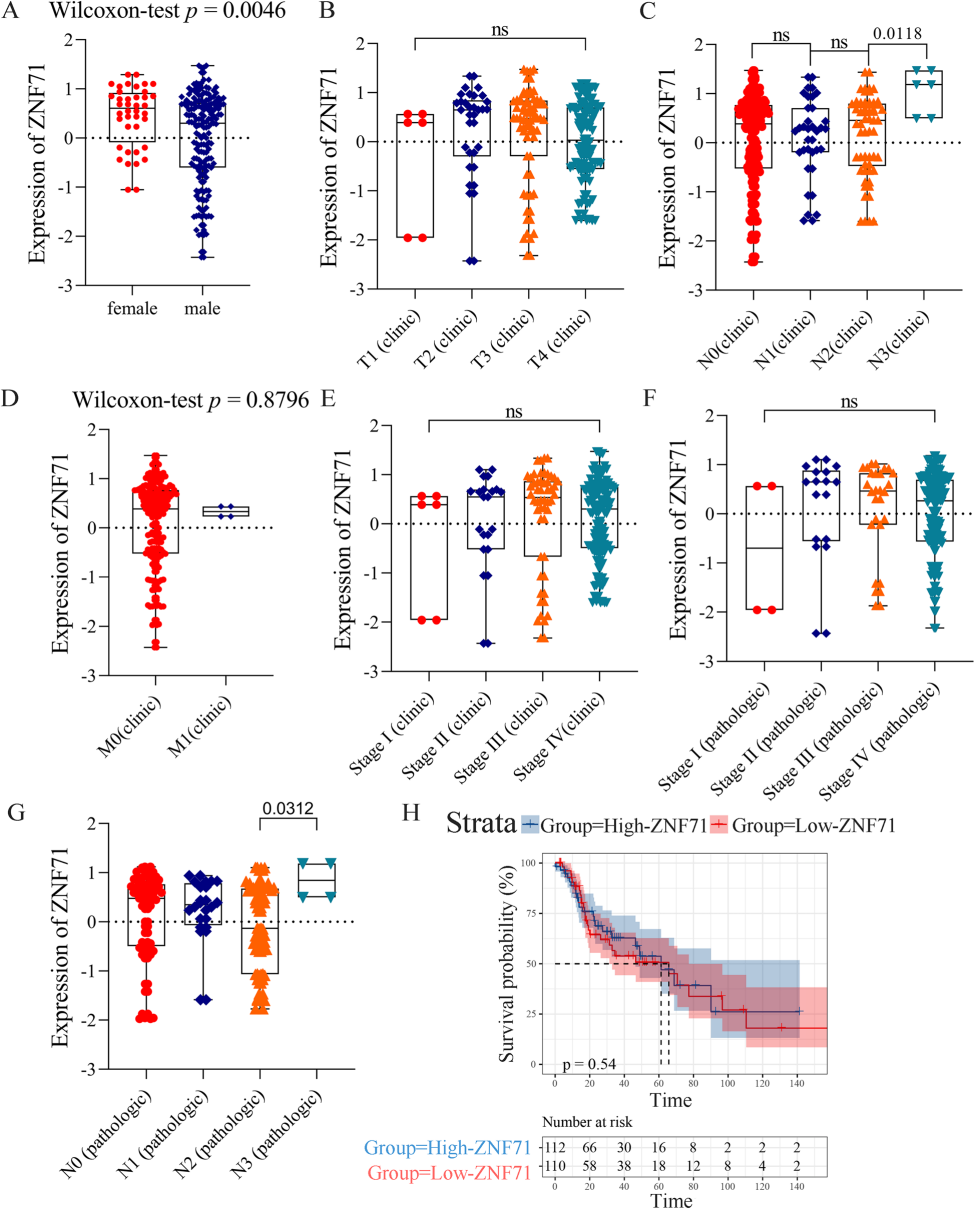
Clinical significance analysis of ZNF71 expression and gender (**A**), T_clinic (**B**), N_clinic (**C**), M_clinic (**D**), stage_clinic (**E**), stage_pathologic (**F**), N_pathologic (**G**), and survival time (**H**)

We also analyzed the relationship between the expression of ZNF71 and clinicopathological parameters of LSCC patients. Male LSCC patients had lower mRNA expression level of ZNF71 than female LSCC patients (*p* = 0.0046, Fig. 4A). But there was no expression difference in non-cancerous samples across genders (*p* > 0.05, Figure S2). Among other clinicopathological parameters, there was no significant difference in the expression of ZNF71 (*p* > 0.05, Fig. 4B–G). And ZNF71 expression was not related to survival time (*p* > 0.05, Fig. 4H).

### ZNF71 was absent in LSCC in different subpopulations based on scRNA-seq analysis

**Fig. 5:**
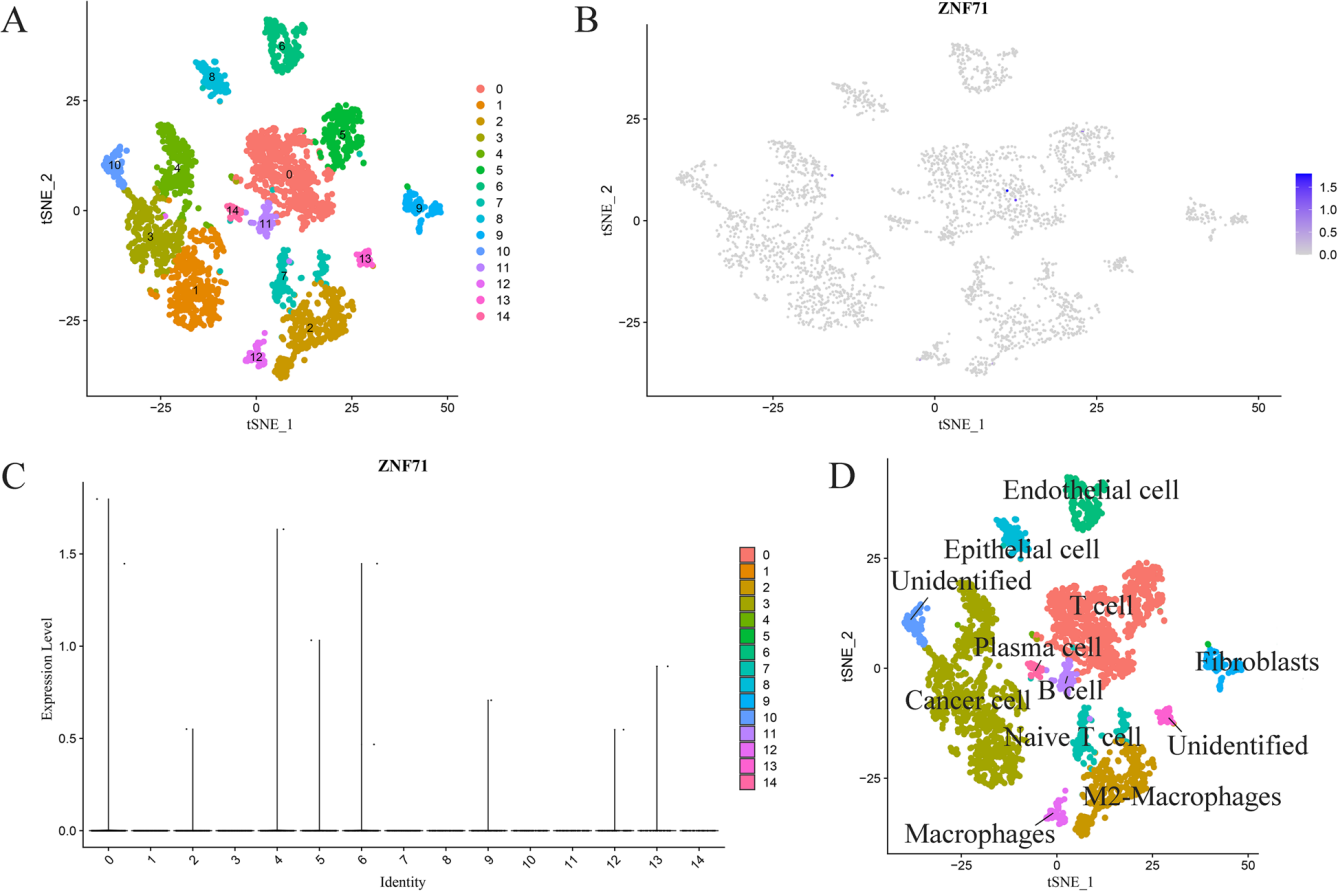
Expression status analysis of ZNF71 based on single cell RNA sequencing data. **A** Dimensionality reduction and clustering of screened single cells. **B** Expression status analysis of ZNF71 in identified clusters. **C** Violin plot of ZNF71 expression in each cluster. **D** Cell type annotation for each cluster

In addition to the protein level and mRNA level, we further explored the expression of ZNF71 in LSCC at the cell level using scRNA-seq data. Firstly, after analyzing the scRNA-seq data and setting appropriate parameters, we divided the cells of LSCC into 15 cell clusters (Fig. 5A). Then, we analyzed and visualized the expression of ZNF71 in these cell clusters with the gene mapping map (Fig. 5B) and violin plot (Fig. 5C). The results showed that there was no expression of ZNF71 in various LSCC cells. Finally, we annotated the cell types according to the marker genes of these cell clusters and found that these cell types are cancer cells, plasma cells, macrophages, M2-macrophages, B cells, T cells, naive T cells, fibroblasts, epithelial cells, endothelial cells, and unidentified cell types (Fig. 5D).

### Potential molecular mechanism of ZNF71 in LSCC

**Fig. 6:**
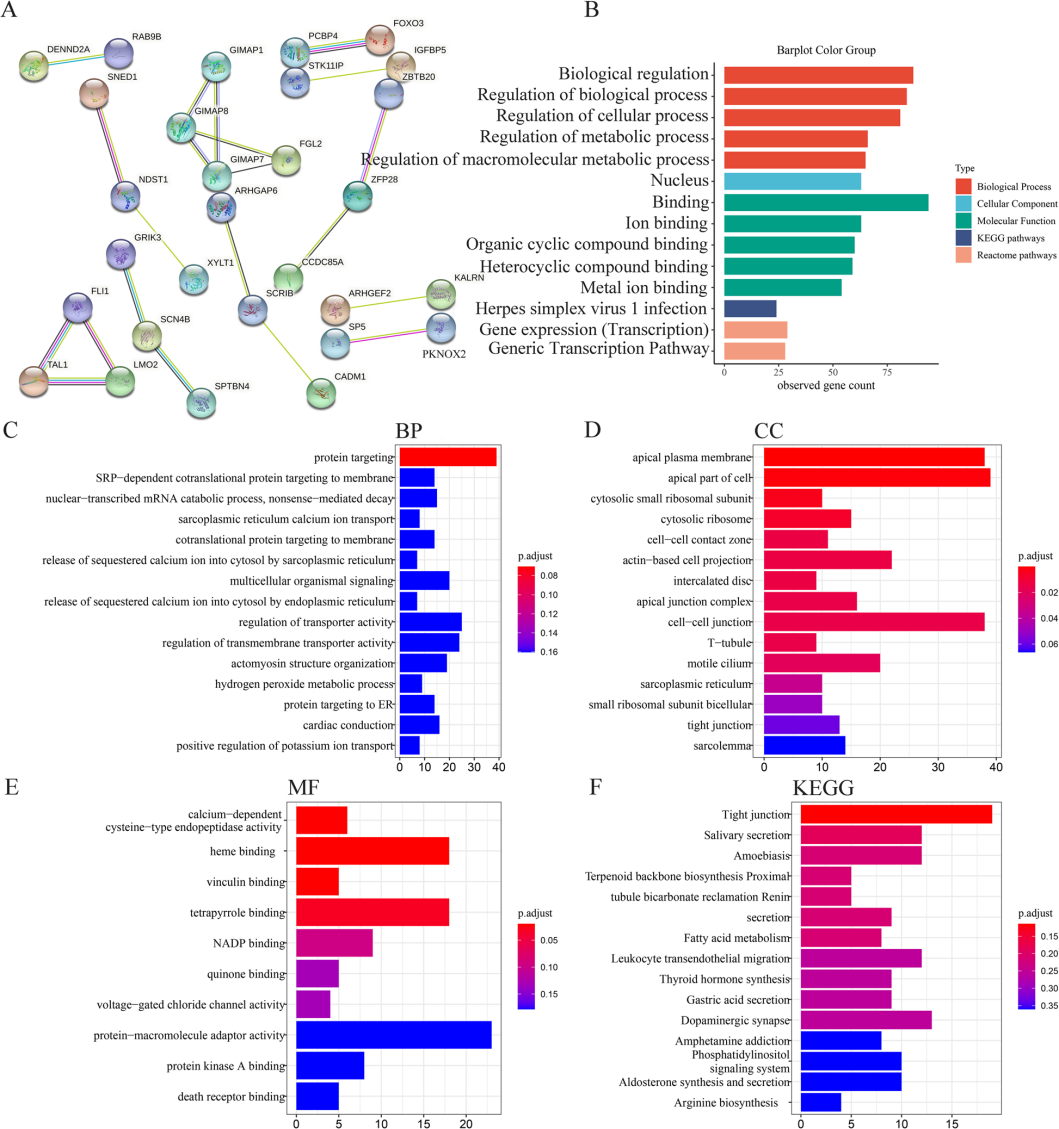
Potential molecular mechanism analysis of ZNF71 in LSCC. **A** Protein-protein interactive network of ZNF71 and its positively and differentially co-expressed genes (PDCEGs). **B** Enrichment analysis of ZNF71 and its PDCEGs. **C**–**F** Biological process (BP), cellular component (CC), molecular function (MF), and KEGG pathway signaling analysis for downstream target genes of ZNF71

To explore the potential molecular mechanism of ZNF71, we constructed a PPI network of ZNF71 and its PDCEGs and conducted enrichment analysis through the STRING website. The interactive relationship is presented in Fig. 6A. These PDCEGs of ZNF71 were mainly enriched in biological regulation, regulation of biological progress, and regulation of cellular progress in the biological process (BP). In cellular components (CC), they concentrated on the nucleus. In terms of molecular function (MF), they were mainly enriched in binding, iron binding, and organic cyclic compound binding. On the Reactome pathway, they were enriched in gene expression and the generic transcription pathway (Fig. 6B).

As ZNF71 is a transcription factor, we further predicted the downstream target genes of ZNF71 through Cistrome DB. We conducted enrichment analysis of ZNF71 and its target genes to explore the potential molecular mechanism of ZNF71 in the occurrence and development of LSCC. In the BP, these genes concentrated on protein targeting (Fig. 6C). In terms of CC, they were mainly enriched in the apical plasma membrane and the apical part of cells (Fig. 6D). On the MF, they were relative to heme binding and tetrapyrrole binding (Fig. 6E). On KEGG signaling pathways, they mainly participated in tight junctions (Fig. 6F). We then further analyzed the expression level of these tight junctions-related genes, and we found they were significantly downregulated in LSCC tissues (Figure S3). And many tight junction-related genes were positively correlated with ZNF71 expression (Table S5).

### The correlation between the expression of ZNF71 and tumor immune infiltration

**Fig. 7:**
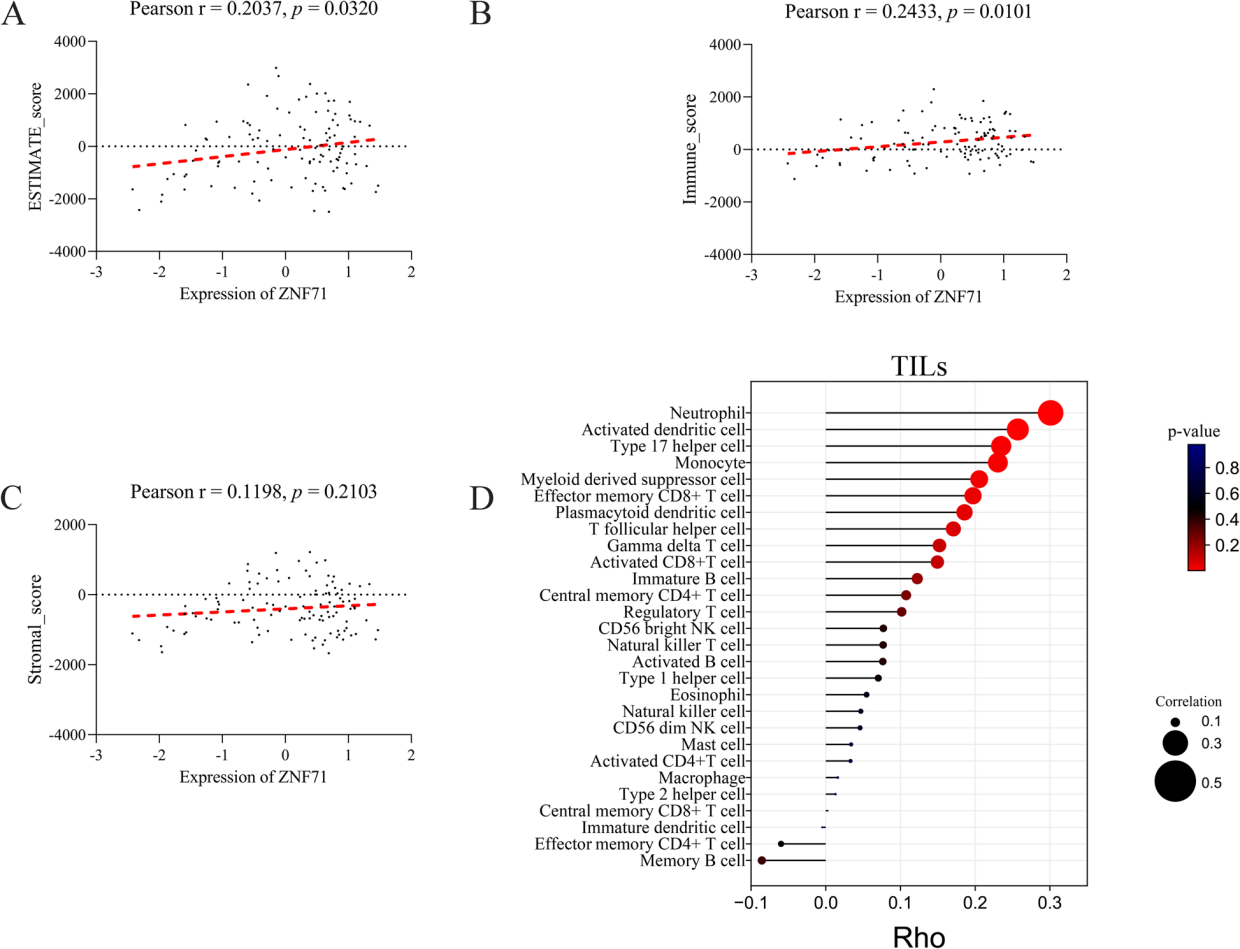
Correlation analysis between ZNF71 expression and ESTIMATE score (**A**), immune score (**B**), stromal score (**C**), and tumor infiltration lymphocytes (TILs, **D**)

We further evaluated the relationship between ZNF71 expression and immune cells and stromal cells in the tumor immune microenvironment (TME) through the ESTIMATE algorithm. The expression of ZNF71 was positively correlated with the ESTIMATE comprehensive score (Pearson *r* = 0.2037, *p* = 0.0320, Fig. 7A). The expression of ZNF71 was positively correlated with immune cell score (Pearson *r* = 0.2433, *p* = 0.0101, Fig. 7B), but not with stromal cell score (Pearson *r* = 0.1198, *p* = 0.2103, Fig. 7C). Furthermore, based on the TISIDB online tool, we further analyzed the relationship between ZNF71 expression and 28 kinds of tumor-infiltrating lymphocytes. The results showed that the expression of ZNF71 was positively correlated with tumor-infiltrating lymphocytes, such as neutrophils, activated dendritic cells, type 17 helper cells, and monocytes (Fig. 7D).

## Discussion

In the present study, we demonstrated that ZNF71 was downregulated in LSCC across in-house IHC staining (LSCC tissue samples = 33; non-cancerous laryngeal tissue samples = 29) and multiple datasets, including gene chips and high-throughput sequencing data (LSCC tissue samples = 313; non-cancerous laryngeal tissue samples = 192) from multi-centers. We found patients with nodal metastasis had lower protein expression level of ZNF71 than patients without nodal metastasis. Then, we observed that the expression of ZNF71 was lower in male LSCC patients than female LSCC patients. Interestingly, according to cancer statistics in 2019, the incidence of laryngeal cancer in male patients was higher than in female patients [19]. Gender is an independent prognostic factor for LSCC patients [20]. Therefore, we speculated that downregulation of ZNF71 is related to the different incidence and pathogenesis of LSCC across genders. Moreover, we found no ZNF71 expression in LSCC samples from different subpopulations through scRNA-seq data analysis. We speculated that the absence of ZNF71 expression in LSCC is related to the pathogenesis of LSCC.

In order to explore the potential molecular mechanism of ZNF71 in LSCC, we conducted enrichment analysis of ZNF71 and its PDCEGs. The results suggest that ZNF71 may interact with its PDCEGs, affecting the transcription process of target genes, and thus may play a role in the occurrence and development of LSCC. Focusing on the function and pathways of ZNF71’s target genes, enrichment analysis showed that ZNF71 may promote the transcription process of membrane proteins and affect the tight junctions between cells. Tight junction proteins have been proven to be involved in epithelial-to-mesenchymal transition and to play an important role in the pathogenesis of some cancers [21, 22]. We found the significant downregulation of these tight junction-related genes in LSCC tissues. Therefore, we speculated that the absence of ZNF71 may promote LSCC development by reducing the tight junctions between tumor cells. Interestingly, our results showed that patients with nodal metastasis had lower protein expression level of ZNF71 than patients without nodal metastasis. We then inferred that reduction of tight junctions associates with nodal metastasis.

Furthermore, we explored the relationship between ZNF71 expression and immune infiltration. According to the results, the expression of ZNF71 was positively correlated with immune cell score, but not with stromal cell score. Hence, we propose that the low expression of ZNF71 means a decrease in immune infiltration, but it does not affect stromal cells. We further calculated which immune cell was positively correlated with the expression of ZNF71. Among those correlative immune cells, neutrophil was most correlated with ZNF71 expression. In TME, neutrophils exert diverse functions [23, 24]. On the one hand, they have been described as a cancer-promoting factor and have been correlated with poor prognosis [23, 24]. Recent studies have revealed that neutrophil extracellular traps released by activated neutrophils mediate the progression of tumors and waken dormant tumor cells [25–27]. On the other hand, they can kill tumor cells as well. Neutrophils can affect tumor growth by orchestrating other immune cells in TME [23]. Overall, neutrophils play complex roles in tumors. Therefore, we speculated that ZNF71 may play a complex role in LSCC by influencing neutrophils. Moreover, it is also important to pay attention to the role of neutrophils in treatment. Previous evidence has shown that neutrophils mediate anti-tumor resistance [24, 28]. In this respect, we assumed that low immune infiltration of neutrophils is not always harmful. Hence, we propose that low expression of ZNF71 does not indicate poor outcome. Interestingly, this is consistent with the results of survival analysis.

Dendritic cells, as professional antigen presenting cells, are key regulators of the immune response within cancers [29, 30]. Moreover, dendritic cells are essential to the activation of effector memory T cells [31]. Interestingly, we observed a positive correlation between ZNF71 expression and these two immune cells. That means LSCC with low expression of ZNF71 has low infiltration of these two immune cells. However, effector memory T cells are crucial in the immune response against tumors [32]. We speculated that the absence of ZNF71 may influence the progression of LSCC by reducing these immune cells. Furthermore, we also found a positive correlation between ZNF71 expression and monocytes. Accumulating evidence has shown that monocytes contribute both to promoting the development of tumors and killing cancers [33, 34]. However, more evidence is needed regarding the function of the low infiltration of monocytes in tumors. Overall, in our results, the low expression of ZNF71 means that the infiltration of most TILs is reduced. TME with low immune infiltration will not only promote the progression of cancer, but also increase the difficulty of therapy [31]. Recent studies by Yan et al. also revealed that immune infiltration is correlated with TNM stages of patients with LSCC [35]. Interestingly, our work revealed that patients with nodal metastasis had lower expression level of ZNF71 than patients without nodal metastasis. Therefore, we speculated that downregulation of ZNF71 is related to immune infiltration and N stage. Taken together, we speculated that the downregulation of ZNF71 may influence the development of LSCC by reducing immune infiltration.

In summary, we demonstrated the downregulation of ZNF71 in LSCC, including different subpopulations of LSCC through data from multi-centers, IHC staining, and scRNA-seq data. We also explored the potential mechanism of ZNF71 in LSCC. The absence of ZNF71 may promote LSCC progression by reducing the tight junction between tumor cells and reducing immune infiltration. However, this study had some limitations. First, analysis between clinicopathologic parameters and the mRNA expression of ZNF71 was limited in TCGA data. Also, the in-house IHC sample size was small. Finally, the exact molecular mechanism of ZNF71 in LSCC requires further experiments in vivo and in vitro.

## Conclusions

Downregulation of ZNF71 was demonstrated by in-house IHC staining and data collected from multi-centers. Also, downregulation of ZNF71 may be related to the different incidence and pathogenesis of LSCC across genders and may also associate with nodal metastasis. Single-cell RNA sequencing analysis also showed the absence of ZNF71 expression in LSCC in different subpopulations. Downregulation of ZNF71 may promote LSCC development by reducing the tight junction between tumor cells and reducing immune infiltration.

## Supplementary Information

**Additional file 1: Table S1.** Raw data for IHC staining.

**Additional file 2: Table S2.** Specific information of included datasets collected from public resources.

**Additional file 3: Table S3.** Expression values of ZNF71 extracted from included datasets.

**Additional file 4: Table S4.** Gene list of positively and differentially co-expressed genes of ZNF71.

**Additional file 5: Table S5.** Pearson correlation analysis between ZNF71 and its putative target genes which relates to tight junction.

**Additional file 6: Figure S1.** Subcellular location of ZNF71 protein (downloaded from HPA website).

**Additional file 7: Figure S2.** Expression difference of ZNF71 across genders.

**Additional file 8: Figure S3.** Expression analysis of ZNF71’s putative target and tight junctions-related genes.

## Data Availability

The following information was supplied regarding data availability: Datasets are available at NCBI GEO and TCGA databases: GSE127165, GSE142083, GSE58911, GSE107591, GSE117005, GSE137308, GSE143224, GSE29330, GSE51985, GSE59102, GSE84957, GSE150321, and TCGA_LSCC data. And the date of immunohistochemistry staining used and/or analyzed during the current study is available at the supplemental materials.

## Abbreviations

LSCC: Laryngeal squamous cell carcinoma
ZNF: Zinc finger protein
TF: Transcription factor
NSCLC: Non-small cell lung cancer
IHC: Immunohistochemistry
ScRNA-seq: Single-cell RNA sequencing
HPA: The Human Protein Atlas
PBS: Phosphate-buffered saline
ROC: Receiver operating characteristic
AUC: Area under the curve
GEO: Gene Expression Omnibus
TCGA: The Cancer Genome Atlas
SRA: Sequence read archive
SMD: Standardized mean difference
sROC: Summary receiver operating characteristic
PDCEGs: Positively and differentially co-expressed genes
DEGs: Differentially expressed genes
PPI: Protein–protein interaction network
DB: Data browser
ChIP-seq: Chromatin immunoprecipitation and DNA sequencing chromatin profiling assays
ATAC-seq: Assay for transposase-accessible chromatin with high throughput sequencing
DNase-seq: DNA affinity purification sequencing
GO: Gene Ontology
KEGG: Kyoto Encyclopedia of Genes and Genomes
95% CI: 95% confidence interval
BP: Biological process
CC: Cellular components
MF: Molecular function
