# Supplementary material for: Downregulation of zinc finger protein 71 in laryngeal squamous cell carcinoma tissues and its potential molecular mechanism and clinical significance: a study based on immunohistochemistry staining and data mining
Source: World J Surg Oncol. 2022 Nov 11;20:359. doi: 10.1186/s12957-022-02823-8 (PMC9650879; doi:10.1186/s12957-022-02823-8)
Supplement: Supplementary file 2 — Additional file 2: Table S2. Specific information of included datasets collected from public resources. [file 12957_2022_2823_MOESM2_ESM.docx]

Table S2. Specific information of included datasets collected from public resources.

| Platforms | Datasets | Last update year | Country | LSCC | Non-LSCC |
| --- | --- | --- | --- | --- | --- |
| GPL20301 | GSE127165 | 2020 | China | 57 | 57 |
| GPL20301 | GSE142083 | 2020 | China | 53 | 53 |
| GPL6244 | GSE58911 | 2018 | USA | 7 | 7 |
| GPL6244 | GSE107591 | 2020 | Italy | 4 | 4 |
| GPL20115 | GSE117005 | 2020 | China | 5 | 5 |
| GPL16791 | GSE137308 | 2019 | China | 6 | 6 |
| GPL5175 | GSE143224 | 2020 | Brazil | 14 | 11 |
| GPL570 | GSE29330 | 2019 | USA | 3 | 5 |
| GPL10558 | GSE51985 | 2019 | China | 10 | 10 |
| GPL6480 | GSE59102 | 2019 | Brazil | 29 | 13 |
| GPL17843 | GSE84957 | 2017 | China | 9 | 9 |
| TCGA | TCGA-LSCC | 2021 | USA | 116 | 12 |

LSCC: laryngeal squamous cell carcinoma.
