# Supplementary material for: Downregulation of zinc finger protein 71 in laryngeal squamous cell carcinoma tissues and its potential molecular mechanism and clinical significance: a study based on immunohistochemistry staining and data mining
Source: World J Surg Oncol. 2022 Nov 11;20:359. doi: 10.1186/s12957-022-02823-8 (PMC9650879; doi:10.1186/s12957-022-02823-8)
Supplement: Supplementary file 6 — Additional file 6: Figure S1. Subcellular location of ZNF71 protein (downloaded from HPA website). [file 12957_2022_2823_MOESM6_ESM.pdf]

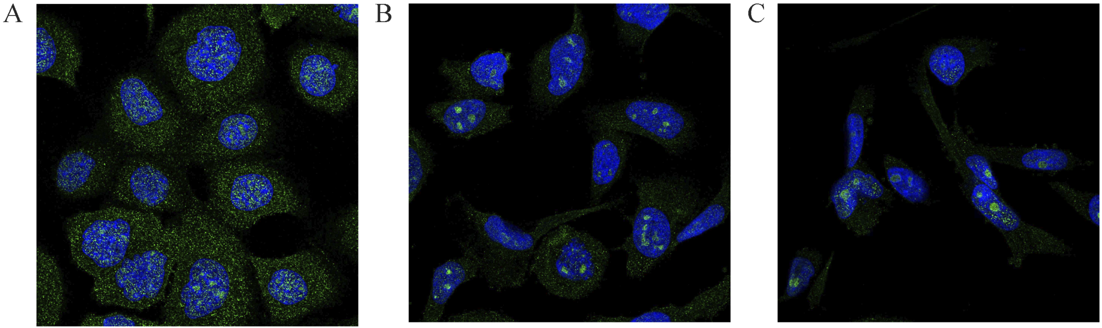

Figure S1. Subcellular location of ZNF71 protein. Images were downloaded from HPA website with number of A-431-HPA018124 (A), U-2 OS-HPA018124 (B), U-251MG-HPA018124 (C).
