# Supplementary figures and images for: Downregulation of zinc finger protein 71 in laryngeal squamous cell carcinoma tissues and its potential molecular mechanism and clinical significance: a study based on immunohistochemistry staining and data mining
Source: World J Surg Oncol. 2022 Nov 11;20:359. doi: 10.1186/s12957-022-02823-8 (PMC9650879; doi:10.1186/s12957-022-02823-8)

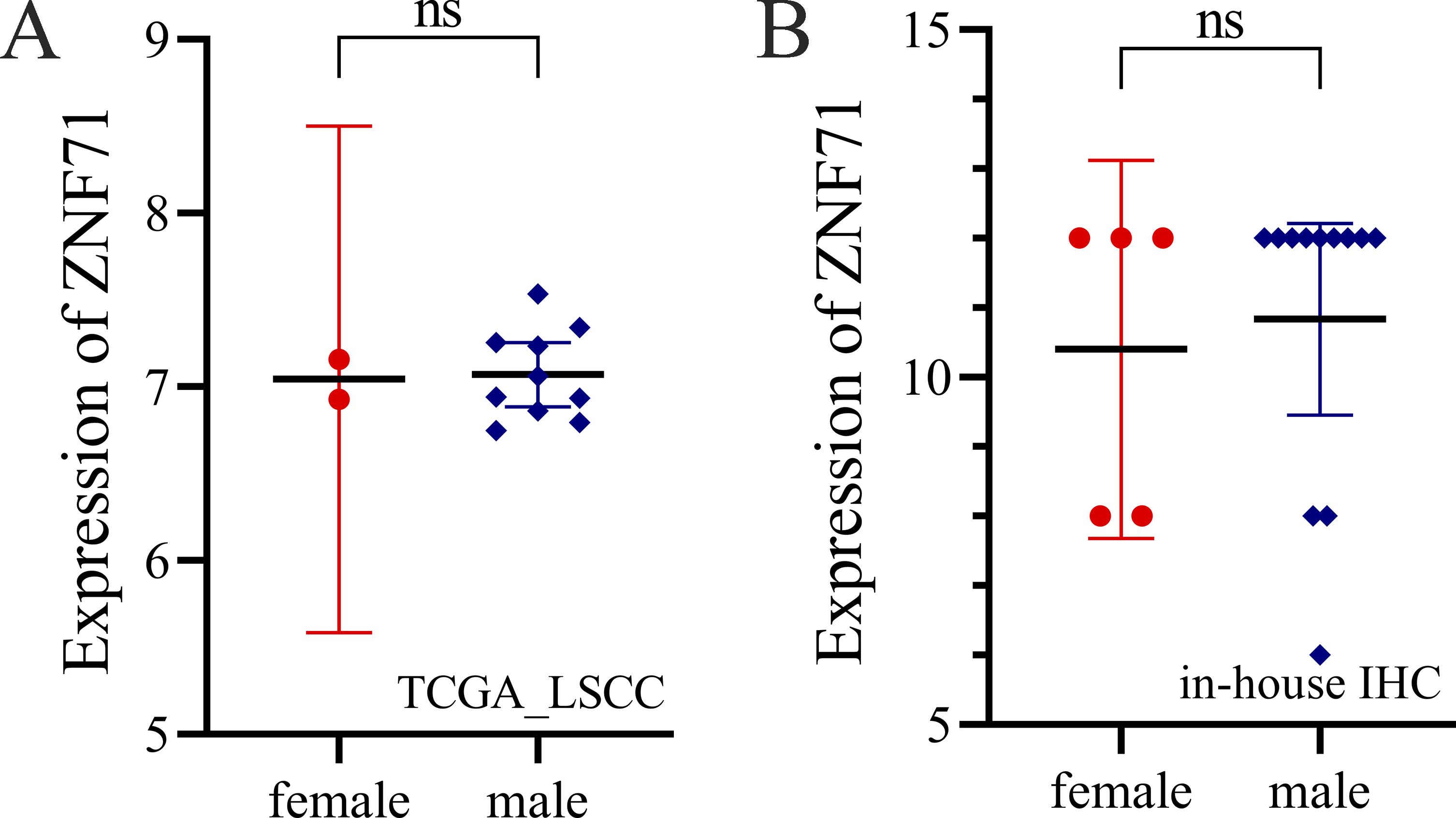

Supplement: Supplementary file 7 — Additional file 7: Figure S2. Expression difference of ZNF71 across genders. [file 12957_2022_2823_MOESM7_ESM.tiff]
